# Conservative management of < 3cm anterior mediastinal lesions in lung cancer screening is safe

**DOI:** 10.1007/s00330-026-12700-7

**Published:** 2026-07-04

**Authors:** Amyn Bhamani, Chuen R. Khaw, Ruth Prendecki, Priyam Verghese, Andrew Creamer, Jennifer L. Dickson, Carolyn Horst, Helen Hall, Sophie Tisi, Monica Mullin, Tanya Patrick, John McCabe, Anne-Marie Hacker, Laura Farrelly, Esther Arthur-Darkwa, Neal Navani, Anand Devaraj, Allan Hackshaw, SUMMIT Consortium, Arjun Nair, Sam M. Janes

**Affiliations:** 1 Lungs for Living Research Centre, UCL Respiratory, University College London, London, UK; 2 Department of Respirology, University of Calgary, Calgary, Canada; 3 Cancer Research UK and UCL Cancer Trials Centre, University College London, London, UK; 4 University College London Hospitals NHS Foundation Trust, London, UK; 5 National Heart and Lung Institute, Imperial College, London, UK; 6 Royal Brompton and Harefield Hospitals, London, UK

**Keywords:** Lung cancer, Lung cancer screening, Low-dose computed tomography, Anterior mediastinal lesions

## Abstract

**Objectives:**

To determine if a pragmatic, conservative approach to managing < 3 cm anterior mediastinal lesions in lung cancer screening (LCS) is safe.

**Materials and methods:**

55- to 77-year-old current or former smokers underwent low-dose computed tomography (LDCT) screening. Anterior mediastinal lesions < 3 cm at baseline were managed conservatively with annual LDCT follow-up for up to 2 years. Lesions ≥ 3 cm at baseline, growing during follow-up (based on visual assessment), or demonstrating concerning radiological characteristics were referred for further assessment. Outcomes for all anterior mediastinal lesions were assessed using follow-up LDCT images, electronic health records and the national cancer registry. Descriptive frequencies were calculated for all reported outcomes.

**Results:**

The baseline prevalence of anterior mediastinal lesions was 0.7% (*n* = 91/12,961). Among 54 participants with < 3 cm lesions who underwent annual LDCT follow-up, 74.1% (*n* = 40/54) completed 2 years of surveillance, while 26.9% (*n* = 14/54) required further assessment due to interval growth. Four participants with growing lesions were diagnosed with thymoma following surgery, and one required adjuvant radiotherapy for an R1 resection margin. Among all participants with anterior mediastinal lesions, 16 underwent surgical resection, resulting in eight thymoma diagnoses. The benign resection rate was 50%. No thymic or anterior mediastinal malignancies have subsequently been diagnosed among participants with < 3 cm lesions who completed surveillance within the study over a median follow-up of 1997.5 days.

**Conclusion:**

Screen-detected anterior mediastinal lesions < 3 cm at baseline without concerning radiological characteristics can be managed conservatively with annual LDCT follow-up. Referral for further assessment only in the event of interval growth does not appear to compromise clinical outcomes.

**Key Points:**

***Question*** *No standardised approach for managing anterior mediastinal lesions identified incidentally through lung cancer screening currently exists. We aimed to determine if utilising a 3 cm baseline diameter referral threshold for screen-detected anterior mediastinal lesions is safe.*

***Findings*** *74% of < 3 cm anterior mediastinal lesions remained stable over two annual screening rounds and did not require a secondary care referral. Four (out of 14) individuals with growing lesions during follow-up were diagnosed with thymoma, and one required adjuvant radiotherapy for an incomplete resection.*

***Clinical relevance*** *Utilising a 3 cm baseline referral threshold is safe and may improve the efficiency of large-scale, population-based LCS by reducing referrals to secondary care.*

**Graphical Abstract:**

**Figure:**
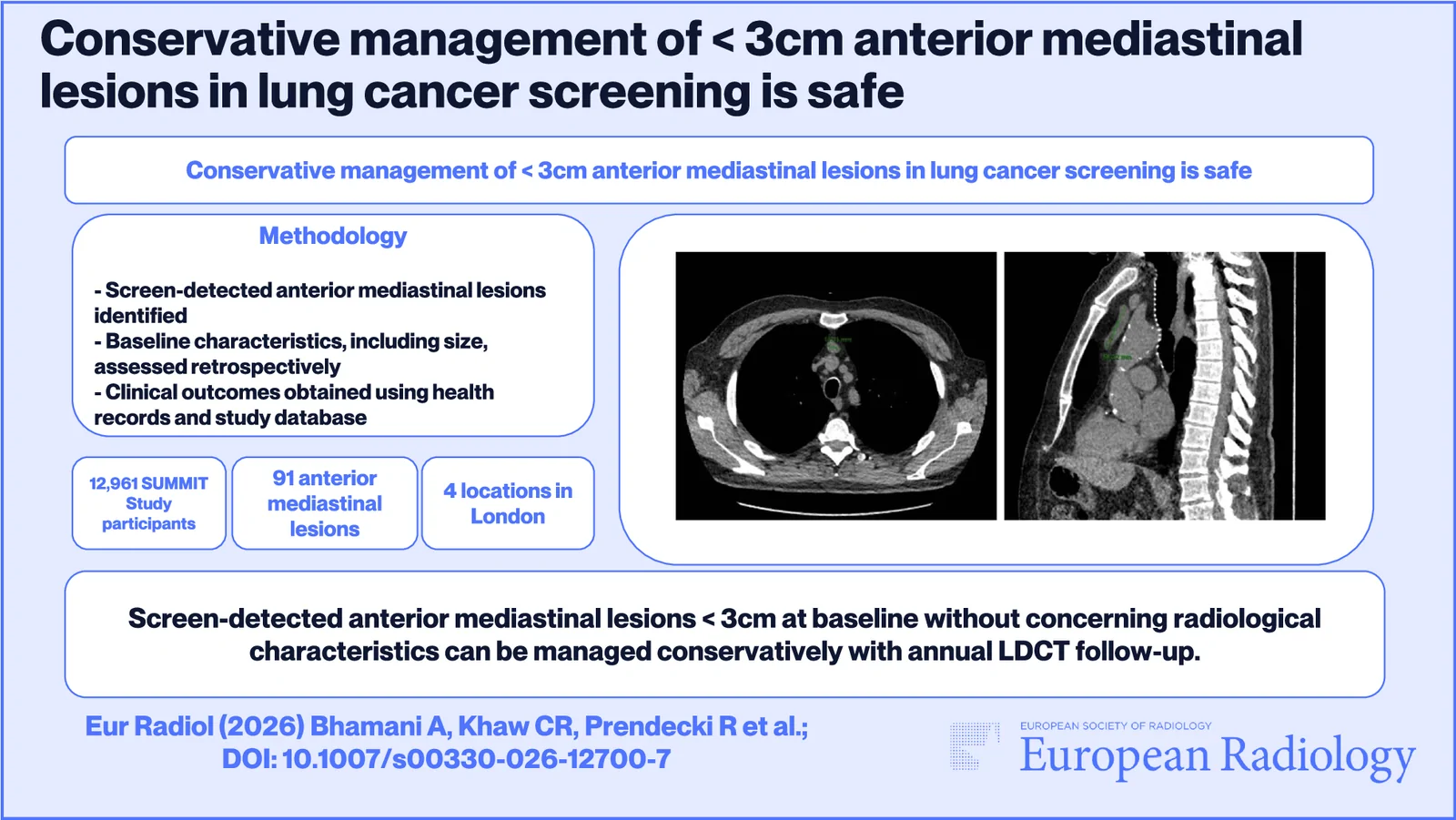

## Introduction

Low-dose computed tomography (LDCT) screening reduces lung cancer-related mortality [1, 2], and a national programme of lung cancer screening (LCS) is now being implemented in the United Kingdom (UK), with full coverage by 2030 [3]. The successful implementation of this programme requires streamlined incidental finding management protocols which minimise over-diagnosis and burden on healthcare providers, while ensuring timely intervention when needed. However, evidence from dedicated screening cohorts to inform such protocols remains insufficient and national recommendations for the management of certain screen-detected incidental findings are largely derived from observational studies of non-screening populations [4].

Although LDCT screening is associated with high sensitivity and specificity for detecting lung cancer [5], image acquisition protocols are not optimised to evaluate incidental extra-parenchymal abnormalities. For example, LDCT is performed without intravenous contrast, limiting its ability to assess and characterise vascular, mediastinal and pleural structures, while greater image noise associated with reduced radiation doses [6] may further limit the evaluation of these structures. Managing extra-parenchymal abnormalities incidentally identified during LCS can, therefore, be challenging.

Determining the optimal management of anterior mediastinal lesions in LCS is particularly difficult because of the relative infrequency with which these are encountered on thoracic imaging. For example, the prevalence of anterior mediastinal lesions in the Framingham Heart Study was 0.9% [7] and 0.4% in the Early Lung Cancer Action Project (ELCAP) LCS cohort [8]. These lesions can also encompass a wide range of differential diagnoses. Although most are thymic in origin, other differentials have varying malignant potential, from benign cysts to lymphoma [9].

In the UK, the national LCS protocol does not recommend a standardised approach towards managing most anterior mediastinal lesions, apart from cystic lesions, which do not require further investigation. Instead, the need for investigations prior to the next screening round is left to the discretion of local screening review and multidisciplinary team (MDT) meetings [4]. In the United States (US), the American College of Radiologists (ACR) white paper recommends further imaging for non-cystic anterior mediastinal lesions, but states that the imaging modality is dependent on the suspected nature of the underlying lesion. In addition, there is no proposed size threshold for further investigations [10]. Considering this, a recent consensus statement from a European Respiratory Society (ERS) task force highlighted the need for further research to help risk-stratify mediastinal lesions [11] and inform protocols for their management.

All anterior mediastinal lesions incidentally identified during the SUMMIT LCS Study were evaluated for size and radiological features [12]. The primary objective of this retrospective analysis was to assess outcomes for participants with anterior mediastinal lesions < 3 cm at baseline to determine if a conservative approach to managing such lesions is safe. Secondary objectives included reporting the overall prevalence of anterior mediastinal lesions in our cohort and assessing outcomes for all individuals referred to secondary care for further investigation.

## Materials and methods

### Ethical approvals and participant consent

The SUMMIT Study (NCT03934866) was a prospective, longitudinal cohort study which aimed to assess the implementation of LDCT screening for lung cancer in a high-risk population and validate a multicancer early detection blood test [5]. Written informed consent was obtained from all study participants. This included permission to access national cancer registry outcomes and follow-up and imaging data for individuals referred to secondary care for further investigation. Additional consent for this retrospective analysis was not required.

Ethical approval was granted by an NHS Research Ethics Committee (17/LO/2004) and the NHS Health Research Authority’s Confidentiality Advisory Group (18/CAG/0054).

### Eligibility criteria for SUMMIT

SUMMIT Study participants were 55–77-year-old (at the time of invitation) current or former smokers from North-East and Central London (NECL) who either met the 2013 United States Preventive Services Task Force (USPSTF) criteria [13] or had a ≥ 1.3% 6-year lung cancer risk based on the Prostate, Lung, Colorectal and Ovarian (PLCO) [14] risk score. Individuals receiving treatment for an active cancer (except adjuvant hormonal treatment) were not eligible [5].

### LDCT acquisition and management protocol for screen-detected anterior mediastinal lesions

Study participants underwent thin collimation (0.625 mm slice thickness), non-contrast LDCT imaging at one of four locations in NECL, each with one CT scanner. Scans were reported by consultant thoracic radiologists according to a protocolised reporting template [12]. Baseline scans required a mandatory comment on the presence of incidental findings within the mediastinum and neck, including anterior mediastinal lesions. Participants with anterior mediastinal lesions ≥ 3 cm in maximum diameter or demonstrating concerning radiological features (irregularity or invasion into surrounding structures) were referred to their local lung cancer MDT for further assessment. Participants with < 3 cm lesions were managed conservatively and followed up within the study with annual LDCT imaging at both their 12-month (Year 1—Y1) and 24-month (Year 2—Y2) appointments. The 3 cm cut-off was based on the observation in ELCAP that thymic lesions smaller than this remained stable or even decreased in size [8].

Follow-up scans required a mandatory comment on any new or changing incidental findings, including those in the mediastinum and neck. Participants with anterior mediastinal lesions demonstrating interval growth or changing morphology (based on visual assessment by the reporting radiologist) during follow-up were referred to their local lung cancer MDT for further assessment.

### Retrospective review of anterior mediastinal lesions

All participants who completed a baseline LDCT between 8th April 2019 and 7th June 2021 were included in this analysis. Scans with reported anterior mediastinal lesions were identified and retrospectively reviewed by a second consultant thoracic radiologist (A.N.—lead radiologist for the SUMMIT Study) who was blinded to the outcome for each lesion. The retrospective review was conducted on standard soft tissue windows (width 400 Hounsfield units (HU), level 40 HU). The maximum diameter for each lesion was measured in anterior-posterior (AP), left-to-right (LR) and cranio-caudal (CC) planes using an electronic calliper, and a ‘mean’ diameter was calculated using the average of the AP and LR (but not CC) measurements, again following the protocol used by ELCAP. Attenuation values (HU) were measured using a region of interest (ROI) chosen carefully to represent the centre and majority of each lesion, while avoiding adjacent structures such as mediastinal fat and bone. Lesions were classified by visual assessment according to baseline features (rounded, triangular, nodular, lobulated, ovoid) and attenuation distribution (homogenous, heterogenous).

### Clinical outcomes for participants with anterior mediastinal lesions

Clinical data for individuals referred to a lung cancer MDT, including details of magnetic resonance imaging (MRI) and positron emission tomography–computed tomography (PET-CT) findings, histological analysis (where available) and follow-up management plans were obtained using electronic health records. Follow-up LDCT imaging for all other individuals was reviewed and assessed for interval anterior mediastinal lesion growth. The quality assured National Cancer Registration and Analysis Service (NCRAS) dataset was used to identify thymic (International Classification of Diseases 10th edition (ICD-10) code C37) and anterior mediastinal malignancies (ICD-10 code C38.1) diagnosed outside the study until 23rd May 2025.

Descriptive frequencies were calculated for these outcome measures. Statistical analysis was carried out on SPSS (version 28.0.0).

## Results

### Cohort characteristics

**Fig. 1:**
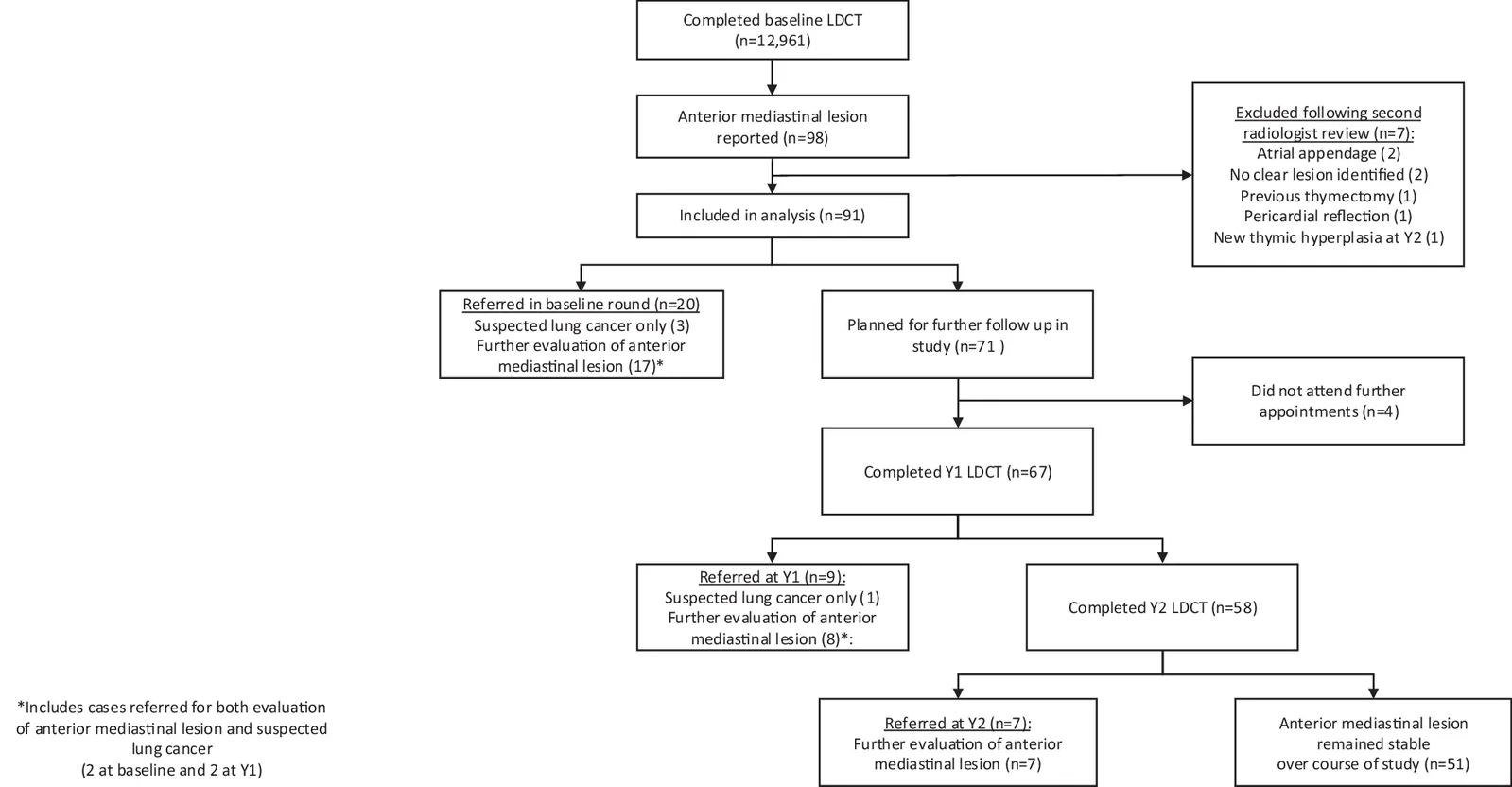
Outcomes for all participants with anterior mediastinal lesions identified through lung cancer screening

12,961 individuals completed a baseline LDCT. Anterior mediastinal lesions had initially been reported in 0.75% (*n* = 98/12,961) of individuals (including 0.12%, *n* = 15/12,961 in whom a lesion was first reported on follow-up imaging but was present on retrospective review of their baseline scan). One participant had undergone a thymectomy for refractory myasthenia gravis 9 months prior to baseline LDCT, which showed residual thymic tissue and was excluded from the analysis. Five additional cases were excluded as no true anterior mediastinal lesion was identified during retrospective review (two atrial appendages, one pericardial reflection and two with no definable lesion), while one participant had no lesion at baseline, but new thymic hyperplasia at Y2. Overall, 91 participants were included in the analysis, a baseline prevalence of 0.7% (91/12,961) (Fig. 1).

56.0% (*n* = 51/91) of these participants were male, and the median age was 66.0 years. Most (89.0%; *n* = 81/91) were of a white ethnic background, and 46.2% (42/91) were currently smoking cigarettes. 16.5% (*n* = 15/91) self-reported a past medical history of cancer (Table 1).

**Table 1:** Characteristics of individuals with anterior mediastinal lesions identified on baseline LDCT (*n* = 91)

	Frequency (*n*)	Percentage (%)
Sex*		
Male	51	56.0
Female	40	44.0
Age*†		
Median (IQR)	66.0 (61.0–72.0)	-
Ethnicity‡		
White	81	89.0
Asian, Black, Mixed, Other	10	11.0
Smoking status‡		
Current smoker	42	46.2
Former smoker	49	53.8
Smoking pack years**		
Median (IQR)	38.0 (30.0–50.0)	-
Previous cancer‡		
Yes	15	16.5
No	76	83.5

* From primary care record

** Calculated at baseline (Y0) based on self-reported smoking history

† Age at time of appointment

‡ Self-reported by participant at baseline (Y0)

### Baseline LDCT characteristics of screen-detected anterior mediastinal lesions

68.1% (*n* = 62/91) of lesions were < 3 cm in maximum diameter and 31.9% (*n* = 29/91) were larger. 69.2% (*n* = 63/91) were largest cranio-caudally (Table 2).

**Table 2:** Baseline LDCT features of anterior mediastinal lesions identified through lung cancer screening (*n* = 91)

	Frequency (*n*)	Percentage (%)
Baseline features		
Lobulated	17	18.7
Nodular	6	6.6
Ovoid	31	34.1
Rounded	28	30.8
Triangular	9	9.9
Maximum diameter (mm)		
< 30	62	68.1
≥ 30	29	31.9
Mean diameter (average of AP and LR) (mm)		
< 30	82	90.1
≥ 30	9	9.9
Maximum diameter plane		
Anterior-posterior (AP)	13	14.3
Left-to-right (LR)	15	16.5
Cranio-caudal (CC)	63	69.2
Attenuation value (HU)		
< 0	14	15.4
0–20	26	28.6
> 20	51	56.0
Attenuation value (HU)		
Median (IQR)	25.0 (6.0–40.0)	-
Attenuation distribution		
Heterogenous	18	19.8
Homogenous	73	80.2

**Fig. 2:**
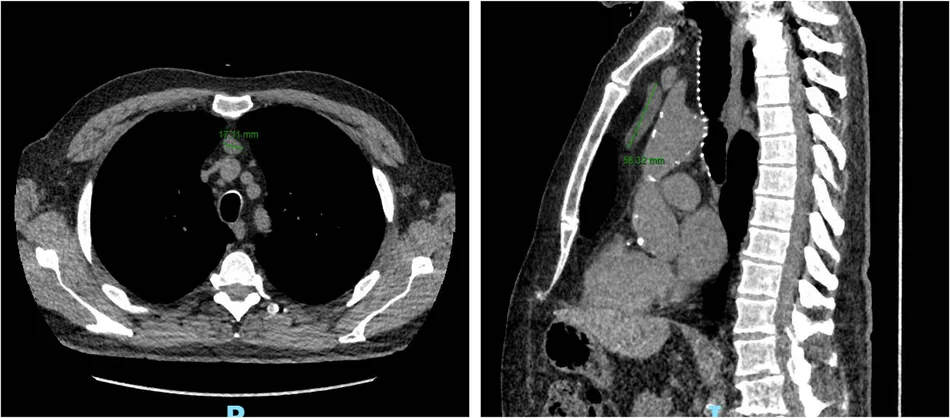
Axial (left) and sagittal (right) views of an anterior mediastinal lesion largest in the cranio-caudal plane

Figure 2 shows a lesion largest cranio-caudally, highlighting the potential for AP and LR measurements alone to significantly underestimate the size of such lesions.

In total, 35.2% (*n* = 32/91) of participants were referred to a lung cancer MDT for further assessment of an anterior mediastinal lesion—18.6% (*n* = 17/91) in the baseline round and 16.5% (*n* = 15/91) at Y1 or Y2. Four individuals did not attend follow-up LDCT imaging appointments.

56.0% (*n* = 51/91) of all participants with anterior mediastinal lesions completed 2 years of annual LDCT follow-up within the study (Table 3).

**Table 3:** Outcomes for all anterior mediastinal lesions identified during the study (*n* = 91)

Outcome	Frequency (*n*)	Percentage (%)
Referred in baseline round for further assessment of anterior mediastinal lesion*	17	18.7
Referred at either Y1 or Y2 for further assessment of growing anterior mediastinal lesion**	15	16.5
Referred for suspected lung cancer (unrelated to anterior mediastinal lesion) in baseline round or Y1	4	5.5
Participant did not attend any subsequent study appointments	4	4.4
Anterior mediastinal lesion remained stable, regressed, or resolved over follow-up in the study	51	56.0

* Includes two participants referred at baseline due to both an anterior mediastinal lesion and suspected lung cancer, and three participants referred after a 3-month nodule follow-up scan due to an anterior mediastinal lesion which had not previously been reported

** Includes two participants referred at Y1 due to both a growing anterior mediastinal lesion and suspected lung cancer

Thoracic MRI and PET-CT were performed in 37.5% (*n* = 12/32) and 50% (*n* = 16/32) of participants referred to a lung cancer MDT, respectively. One participant underwent both types of imaging.

50% (*n* = 16/32) of participants referred to a lung cancer MDT underwent surgical resection. 34.4% (*n* = 11/32) were kept under imaging surveillance (*n* = 8), discharged following a referral at Y2 (*n* = 1) or referred back to the study for ongoing LDCT follow-up (*n* = 2).

These outcomes are summarised in Supplementary Table 1.

### Tissue diagnoses following surgical resection and LDCT characteristics of confirmed thymomas

Surgical resection confirmed thymoma in 50% (*n* = 8/16) of participants. The remaining 50% (*n* = 8/16) were benign lesions: 62.5% (*n* = 5/8) of these were thymic or bronchogenic cysts, while the remaining cases (37.5%, *n* = 3/8) were a thyroid goitre, a thymolipoma, and a reactive lymph node (Table 4).

**Table 4:** Tissue diagnoses for anterior mediastinal lesions surgically resected following a lung cancer multidisciplinary team referral

No	Referral timepoint	Age	Gender	Maximum diameter (mm)	Mean diameter* (mm)	Shape	Attenuation value	Attenuation distribution	Longest axis plane	PET SUV max	MRI diagnosis	Tissue diagnosis
1	Baseline	64	Female	47.3	36.1	Lobulated	45	Homogenous	CC	N/A	Inconclusive	Thymoma
2	Baseline	64	Male	37.4	23.2	Triangular	10	Homogenous	CC	N/A	Complex thymic lesion	Thymic cyst
3	Baseline	71	Female	58.7	49.3	Lobulated	42	Homogenous	CC	2.6	Malignant lesion	Thymoma
4	Baseline	73	Male	31.1	21.4	Lobulated	48	Homogenous	CC	N/A	N/A	Bronchogenic cyst
5	Baseline	59	Female	45.5	36.2	Lobulated	57	Homogenous	LR	1.9	N/A	Multinodular goitre
6	Baseline	58	Female	39.0	24.2	Ovoid	24	Heterogenous	CC	0	Thymoma	Thymic cyst
7	Baseline	73	Female	92.8	49.2	Triangular	6	Homogenous	CC	N/A	Thymic cyst	Thymic cyst
8	Baseline	65	Male	25.1	15.3	Ovoid	31	Homogenous	CC	3.6	N/A	Reactive lymph node
9	Baseline	59	Male	67.0	35.4	Lobulated	36	Heterogenous	CC	4.9	N/A	Thymoma
10	Baseline + 3 months	69	Male	77.0	23.3	Lobulated	40	Homogenous	CC	3.8	N/A	Thymoma
11	Year 1	72	Male	26.1	20.4	Rounded	59	Homogenous	CC	3.9	N/A	Thymoma
12	Year 1	75	Female	33.2	23.5	Ovoid	−8	Heterogenous	CC	N/A	Cystic lesion	Thymic cyst
13	Year 1	63	Female	18.0	14.8	Lobulated	45	Homogenous	AP	N/A	N/A	Thymoma
14	Year 1	58	Male	21.8	16.2	Lobulated	30	Homogenous	CC	4.4	N/A	Thymoma
15	Year 2	72	Female	7.5	5.4	Rounded	1	Homogenous	CC	0	N/A	Thymolipoma
16	Year 2	55	Female	10.5	8.5	Rounded	53	Homogenous	CC	N/A	N/A	Thymoma

* Mean diameter refers to the average of AP and LR diameters

87.5% (*n* = 7/8) of confirmed thymomas appeared homogenous on LDCT, and all were either rounded or lobulated. Only one was heterogenous and required referral for further assessment at baseline (maximum diameter 67.0 mm).

**Fig. 3:**
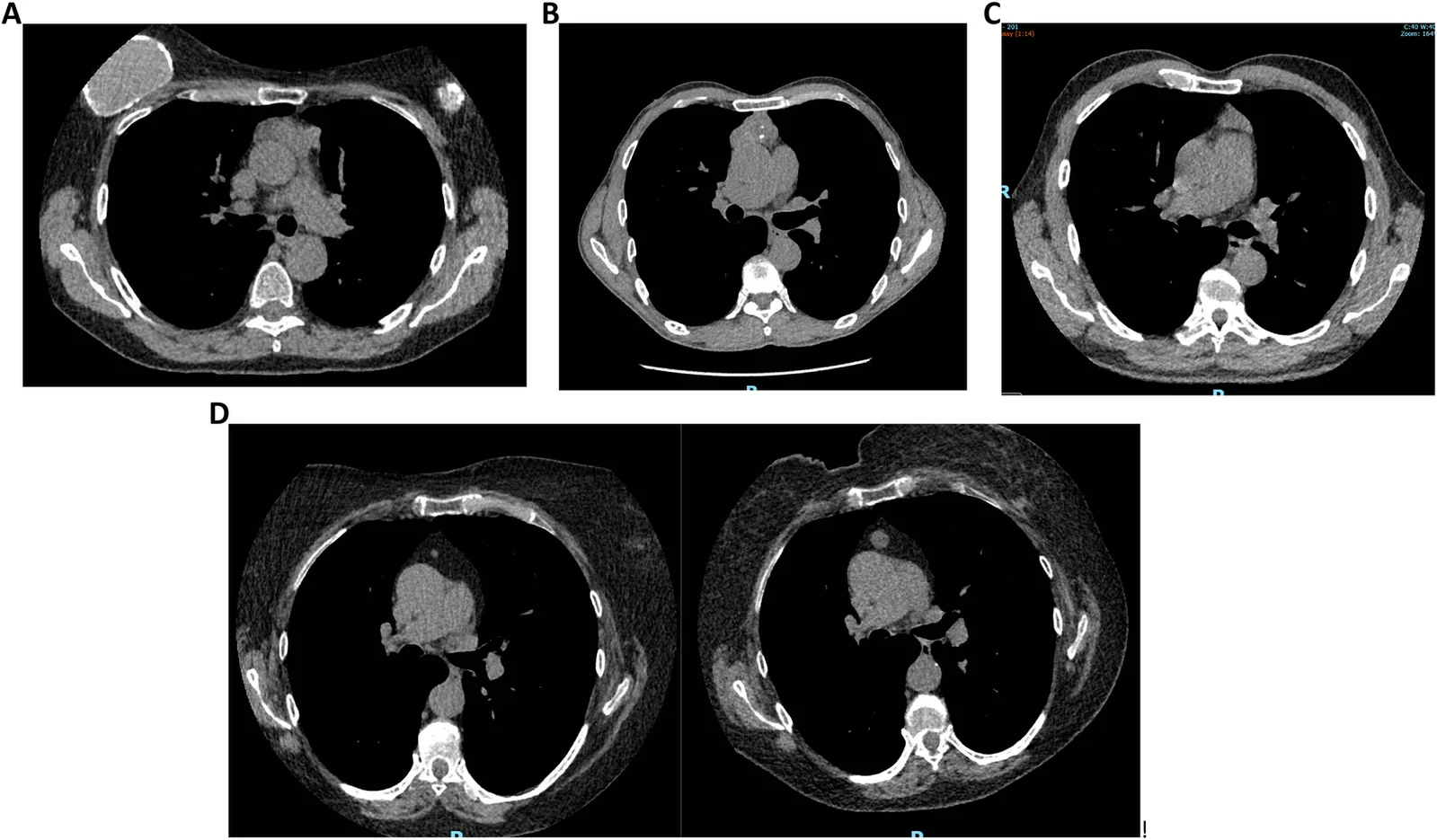
**A** Axial LDCT image on mediastinal window showing a lobulated, homogenous lesion, confirmed to be a type B2 thymoma following surgical resection. **B** Axial LDCT image on mediastinal window showing a lobulated, heterogenous (due to the presence of internal calcification) lesion, confirmed to be a type A thymoma following surgical resection. **C** Axial LDCT image on mediastinal window showing a triangular, homogenous lesion confirmed to be a thymic cyst following surgical resection. **D** Axial LDCT images on mediastinal windows showing a 7.5 mm (cranio-caudal) rounded, homogenous lesion at baseline (left) growing to 14.8 mm after 2 years (right). Attenuation value was 1 HU, and surgical resection confirmed a benign thymolipoma

Figure 3 shows LDCT images of selected anterior mediastinal lesions with confirmed tissue diagnoses, while Supplementary Table 2 compares the baseline LDCT characteristics of histologically confirmed thymomas and benign anterior mediastinal lesions.

37.5% (*n* = 3/8) of thymomas confirmed following surgical resection were high-risk (Type B2). These lesions were either ≥ 3 cm at baseline or demonstrated interval growth on follow-up LDCT. Two patients required adjuvant radiotherapy for incomplete resection margins—one was referred at baseline due to a ≥ 3 cm lesion, while the other was referred after interval growth on their Y1 scan (Table 5).

**Table 5:** Histology and clinical outcomes for the eight screen-detected thymomas excised following a lung cancer multidisciplinary team referral

Referral timepoint	Age	Maximum diameter (mm)	Mean diameter* (mm)	Thymoma type	Masaoka-Koga stage	TNM stage	Resection margin	Adjuvant treatment required
Baseline	64	47.3	36.1	B2	IIB	Not reported	R1	Radiotherapy
Baseline	71	58.7	49.3	A	IIA	pT1a N0	R0	Nil
Baseline	59	67.0	35.4	A	IIA	pT1a N0	Not available	Nil
Baseline + 3 months	69	77.0	23.2	B2	Not reported	pT1a Nx	R0	Nil
Year 1	58	21.8	16.2	AB	I	Not reported	R0	Nil
Year 1	72	26.1	20.4	AB	IIA	pT1a N0	R1	Radiotherapy
Year 1	63	18.0	14.8	B2	Not reported	PT1a N0	R0	Nil
Year 2	55	10.5	8.5	Micronodular thymoma with lymphoid stroma	Not reported	pT1	R0	Nil

* Mean diameter refers to the average of AP and LR diameters

### Analysis of 3 cm maximum diameter threshold for referral at baseline

**Fig. 4:**
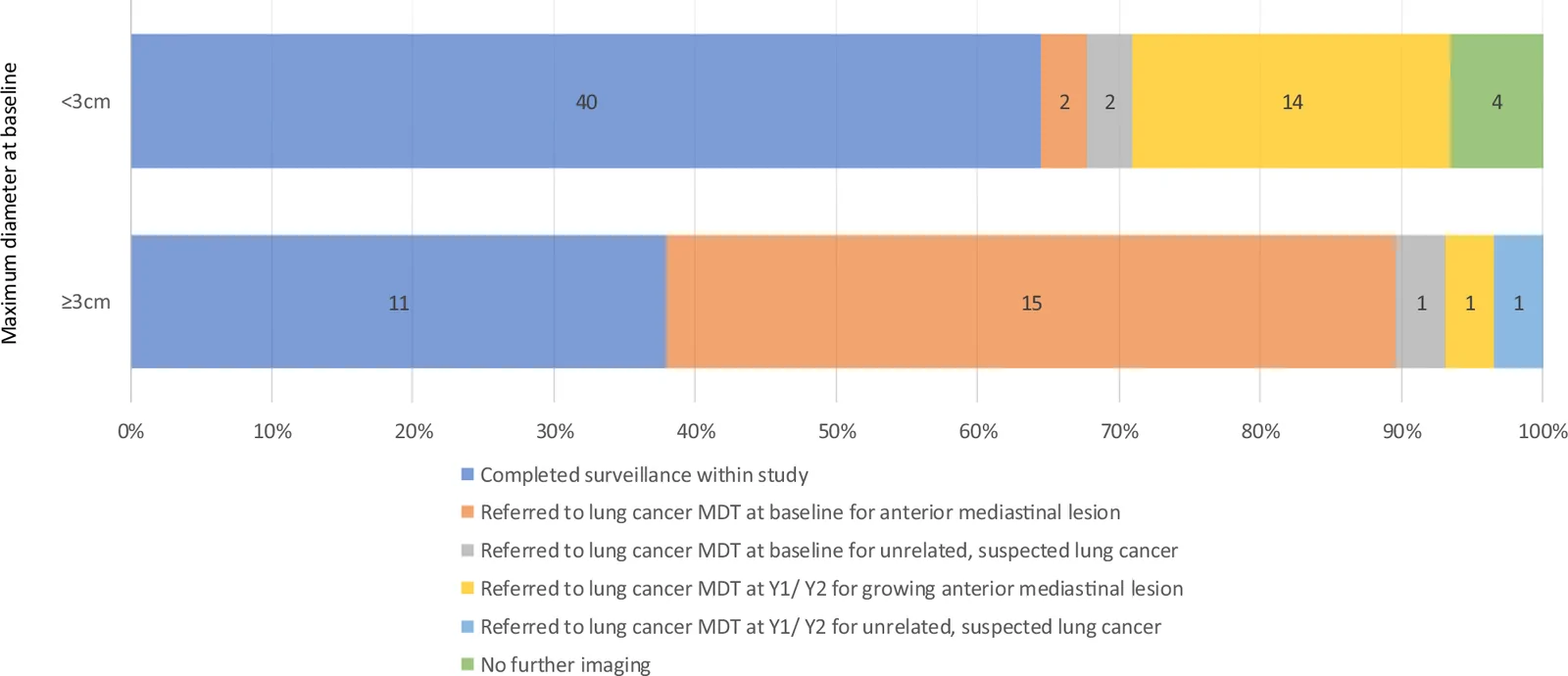
Outcomes for all participants with anterior mediastinal lesions identified through lung cancer screening, stratified by maximum baseline diameter

Outcomes for participants with anterior mediastinal lesions, stratified by maximum diameter at baseline, are presented in Fig. 4.

68.1% (*n* = 62/91) of participants had < 3 cm anterior mediastinal lesions at baseline. 6.5% (*n* = 4/62) of these were referred to a lung cancer MDT; however, two of these referrals were due to suspected lung cancer (unrelated to the anterior mediastinal lesion), while one lesion was initially reported as ≥ 3 cm but found to be < 3 cm on retrospective review. One referral was prompted by the presence of a co-existing unilateral pleural effusion. Overall, 93.5% of < 3 cm anterior mediastinal lesions (*n* = 58/62) were planned for follow-up within the study.

6.9% (4/58) of participants with planned study follow-up did not attend further appointments. Amongst the remaining individuals, 25.9% (*n* = 14/54) required a lung cancer MDT referral after their Y1 or Y2 scan showed a growing lesion. 74.1% (*n* = 40/54) completed surveillance within the study and did not require a referral (median follow-up duration 737.5 days).

28.6% (*n* = 4/14) of individuals referred to a lung cancer MDT due to a growing lesion were diagnosed with thymoma after surgical resection—one required adjuvant radiotherapy for an R1 resection margin. 21.4% (*n* = 3/14) of other participants were diagnosed with non-thymic malignancy; however, one was already under clinical follow-up for thyroid cancer, another had co-existent progressive axillary lymphadenopathy and was diagnosed with lymphoma, and the third had a concurrent, new, 19 mm lung nodule and was subsequently diagnosed with cancer of unknown primary.

Over a median follow-up of 1997.5 days, no thymic or anterior mediastinal malignancies were diagnosed outside the study amongst participants who were not referred to a lung cancer MDT for further assessment.

## Discussion

In this cohort of LCS participants, the prevalence of screen-detected anterior mediastinal lesions was 0.7%, similar to that reported in previous studies [8, 15]. Most lesions were < 3 cm at baseline; nearly three-quarters of these remained stable over two annual screening rounds and did not require a secondary care referral. Four out of 14 participants referred due to a growing lesion on follow-up imaging were diagnosed with thymoma, and one required adjuvant radiotherapy due to an incomplete surgical resection margin. The benign resection rate amongst participants who underwent surgery was 50%.

Our analysis highlights several key points. First, these data validate a 3 cm baseline referral threshold for screen-detected anterior mediastinal lesions by demonstrating that, in the absence of concerning CT features, lesions < 3 cm can be kept under clinically indicated imaging follow-up within screening programmes without compromising on clinical outcomes. Conversely, the presence of features such as irregular margins, unequivocal heterogeneity, encasement or infiltration of surrounding structures and co-existent lymphadenopathy should prompt secondary care referral regardless of size, as these may be indicative of aggressive lesions such as thymic carcinoma or lymphoma. Similarly, co-existing pleural or pericardial effusions or pleural nodularity are concerning for drop metastases and also warrant urgent assessment [16, 17]. However, it is reassuring that only one participant in our cohort with a < 3 cm anterior mediastinal lesion was referred to secondary care in the baseline round due to the presence of a unilateral pleural effusion (ultimately unrelated to the lesion), and no referrals were made due to suspicious morphology alone. This is in keeping with the generally indolent nature of thymic tumours and the relative rarity of invasive thymoma or thymic carcinoma [18].

Our findings build on data reported by ELCAP, in which most < 3 cm thymic lesions remained stable or regressed over a 1-year interval, and show that a conservative, ‘observational’ approach towards managing these lesions remains safe, even when the duration of follow-up is extended. The biennial nature of LCS in the UK will also enable longer-term, opportunistic follow-up of these lesions, although a small proportion of individuals who ‘age out’ may require a secondary care referral for further surveillance [19].

These data may also be applied to ‘non-screening’ clinical cohorts, where anterior mediastinal lesions are usually referred for surgical resection. In this setting, the absence of concerning CT features may provide reassurance that such lesions can be kept under careful surveillance, particularly in individuals at higher risk for surgery (e.g., due to competing co-morbidities or poor performance status) or those reluctant to undergo surgical resection without prior tissue confirmation of malignancy.

Second, there was significant heterogeneity in management plans recommended by lung cancer MDTs following a referral from the study. Highlighting this, thoracic MRI and PET-CT were performed in 37.5% and 50% of cases, respectively, while one individual underwent both types of imaging. In addition, 50% of surgical resections confirmed a benign lesion, comparable to data reported by a large Korean screening cohort showing a 77% benign resection rate [15]. These findings reflect ‘real-world’ practice and demonstrate how the optimal management of anterior mediastinal lesions remains uncertain. While a standardised algorithm for investigating such lesions (in which morphologic and attenuation characteristics are key in determining subsequent investigations) has recently been proposed [19], our data highlights the need for dedicated protocols to cover the management of lesions identified through LCS, where these are most likely to be isolated and discovered incidentally.

Third, most anterior mediastinal lesions were largest in the cranio-caudal plane and required review of sagittal images to enable an accurate assessment of their size. Our findings validate a prior analysis of normal thymic morphology from the 1980s, which showed that the longest thymic measurements were cranio-caudal in all age groups [20]. This includes 87.5% (*n* = 7/8) of thymomas confirmed following surgical resection, highlighting the importance of assessing such lesions in both axial and sagittal planes to avoid underestimation of size; indeed, several lesions ≥ 3 cm at baseline were underestimated and, therefore, not immediately referred for further assessment, presumably because the radiologist omitted the review of the sagittal plane on these occasions. It is also important to note that the ‘diameter’ reported in the original ELCAP analysis referred to an average of axial length and width, but not height, of lesions. Applying these criteria to our cohort would also have captured all three thymomas diagnosed immediately after a baseline scan. Although this may suggest that axial measurements alone are sufficient, the small sample size precludes definitive conclusions.

Incidental finding management in LCS remains challenging due to the relative paucity of data from dedicated screening cohorts. Significant geographical variations in practice exist, and there remains a need to standardise management across programmes. SUMMIT is one of the largest LCS cohorts in Europe, and our analysis provides much-needed evidence to inform national and international guidelines, demonstrating that conservative management of screen-detected anterior mediastinal lesions < 3 cm at baseline is safe. Notably, we did not set out to prove that this threshold would differentiate between benign and malignant lesions. Rather, we aimed to show that this was a pragmatic strategy intended to reduce unnecessary referrals to secondary care without compromising on clinical outcomes. This approach aligns with pulmonary nodule management guidelines, which recommend CT surveillance for sub-solid or small solid nodules in the first instance, even though a proportion of these represent small lung cancers.

This analysis has acknowledged limitations. Despite the size of SUMMIT, the number of anterior mediastinal lesions was small, and a gold standard tissue diagnosis was only available for a minority (17%) of participants. While multivariable regression analyses could potentially identify factors associated with anterior mediastinal malignancies, these analyses would be unreliable with only 16 histological diagnoses and 8 confirmed thymomas. Pooled analyses of national LCS programme data would provide an opportunity to validate these results in a larger cohort. In addition, the duration of imaging follow-up within the study was relatively short, considering the natural history of thymomas. However, to date, no thymic or anterior mediastinal tumours have been diagnosed amongst participants not referred for further investigation by the study, according to national registry data. Finally, imaging characteristics (including morphology and growth) were assessed subjectively, and inter-observer variability was not quantified in the interest of pragmatism. However, no clear definition of growth in anterior mediastinal lesions exists and ‘double reading’ of LDCT imaging in large-scale, population-based LCS is unlikely to be feasible. Our approach, therefore, reflects ‘real-world’ practice.

In summary, our analysis suggests that, in the absence of concerning LDCT features, a 3 cm baseline diameter referral threshold for screen-detected anterior mediastinal lesions is safe, enabling most participants to continue surveillance within the screening programme without compromising on clinical outcomes. The utilisation of a size-based referral threshold may improve the efficiency of a national LCS programme and reduce unnecessary referrals to secondary care.

## Supplementary information

ELECTRONIC SUPPLEMENTARY MATERIAL

Appendix 1 - SUMMIT consortium member names and affiliations

## Abbreviations

ACR: American College of Radiologists
AP: Anterior-posterior
CC: Cranio-caudal
ELCAP: Early Lung Cancer Action Project
ERS: European Respiratory Society
HU: Hounsfield units
ICD-10: International Classification of Diseases–10th edition
LCS: Lung cancer screening
LDCT: Low-dose computed tomography
LR: Left-to-right
MDT: Multidisciplinary team
MRI: Magnetic resonance imaging
NECL: North-East and Central London
PET-CT: Positron emission tomography–computed tomography
UK: United Kingdom
USPSTF: United States Preventive Services Task Force
Y1: Year 1
Y2: Year 2
