# Supplementary material for: Conservative management of < 3cm anterior mediastinal lesions in lung cancer screening is safe
Source: Eur Radiol. 2026 Jul 4;36(11):8225–35. doi: 10.1007/s00330-026-12700-7 (PMC13661742; doi:10.1007/s00330-026-12700-7)
Supplement: Supplementary file 1 [file 330_2026_12700_MOESM1_ESM.pdf]

ELECTRONIC SUPPLEMENTARY MATERIAL

Conservative management of <3cm anterior mediastinal lesions in lung cancer screening is safe

Supplementary Table 1: Outcomes for all anterior mediastinal lesions referred to a lung cancer multidisciplinary team for further assessment (n=32)

Outcome	Frequency (n)	Percentage (%)
Resected	16	50.0
Kept under imaging surveillance or discharged	9	28.1
Referred back to SUMMIT – remained stable over follow-up in study	2	6.3
Known thyroid cancer – presumed metastasis	1	3.1
Other*	4	12.5

*Includes: Lung adenocarcinoma (unrelated to anterior mediastinal lesion), lymphoma, cancer of unknown primary and ectopic thyroid (clinical diagnosis)

Supplementary Table 2: Baseline LDCT characteristics of all thymomas and benign anterior mediastinal lesions confirmed following surgical resection

	Thymomas (n, %)	Benign (n, %)
Baseline features		
Lobulated	6 (75%)	1 (12.5%)
Rounded	2 (25%)	1 (12.5%)
Ovoid	0 (0%)	3 (37.5%)
Rounded	0 (0%)	1 (12.5%)
Triangular	0 (0%)	2 (25%)
Maximum diameter (mm)		
<30	4 (50%)	2 (25%)
≥30	4 (50%)	6 (75%)
Mean diameter (average of AP and transverse) (mm)		
<30	5 (62.5%)	6 (75%)
≥30	3 (37.5%)	2 (25%)
Maximum diameter plane		
Anterior-posterior (AP)	1 (12.5%)	1 (12.5%)
Cranio-caudal (CC)	7 (87.5%)	7 (87.5%)
Attenuation value (HU)		
<0	0 (0%)	1 (12.5%)
0-20	0 0%)	3 (37.5%)
>20	8 (100%)	4 (50%)
Attenuation value (HU)		
Median (IQR)	43.5 (37.0 – 51.0)	17.0 (2.25 – 43.75)
Attenuation distribution		
Heterogenous	1 (12.5%)	2 (25%)
Homogenous	7 (87.5%)	6 (75%)
