# Supplementary material for: Conservative management of < 3cm anterior mediastinal lesions in lung cancer screening is safe
Source: Eur Radiol. 2026 Jul 4;36(11):8225–35. doi: 10.1007/s00330-026-12700-7 (PMC13661742; doi:10.1007/s00330-026-12700-7)
Supplement: Supplementary file 2 [file 330_2026_12700_MOESM2_ESM.docx]

**Appendix 1 - SUMMIT consortium member names and affiliations**

| Aashna Samson | University College London Hospitals NHS Foundation Trust, London |
| --- | --- |
| Adam Szmul | Satsuma Lab, UCL Hawkes Institute |
| Adefiola Olabode | University College London Hospitals NHS Foundation Trust, London |
| Alan Shaw | Whittington Health NHS Trust, London |
| Alberto Villanueva | University College London Hospitals NHS Foundation Trust, London |
| Ali Mohammed | Barts Health NHS Trust, London |
| Alice Cotton | University College London Hospitals NHS Foundation Trust, London |
| Allan Hackshaw | CRUK & UCL Cancer Trials Centre, University College London, London |
| Amyn Bhamani | Lungs for Living Research Centre, UCL Respiratory, University College London, London |
| Anand Devaraj | National Heart and Lung Institute, Imperial College, London  Royal Brompton and Harefield Hospitals, London |
| Anant Patel | Royal Free London NHS Foundation Trust, London |
| Andrew Creamer | Lungs for Living Research Centre, UCL Respiratory, University College London, London |
| Andrew Crossingham | University College London Hospitals NHS Foundation Trust, London |
| Andrew Perugia | Noclor Research Support, London |
| Angshu Bhowmik | Homerton University Hospital Foundation Trust, London |
| Anna Sikorska | University College London Hospitals NHS Foundation Trust, London |
| Anne-Marie Hacker | CRUK & UCL Cancer Trials Centre, University College London, London |
| Anthony Edey | University College London Hospitals NHS Foundation Trust, London |
| Antonette Andrews | University College London Hospitals NHS Foundation Trust, London |
| April Neville | University College London Hospitals NHS Foundation Trust, London |
| Arjun Nair | University College London Hospitals NHS Foundation Trust, London |
| Burcu Ozaltin | Satsuma Lab, Centre for Medical Image Computing (CMIC), London |
| Camilla Mitchell | University College London Hospitals NHS Foundation Trust, London |
| Carolyn Horst | Lungs for Living Research Centre, UCL Respiratory, University College London, London |
| Catherine Nestor | University College London Hospitals NHS Foundation Trust, London |
| Celia Snell | University College London Hospitals NHS Foundation Trust, London |
| Charlie Sayer | University College London Hospitals NHS Foundation Trust, London |
| Charlotte Cash | Royal Free London NHS Foundation Trust, London |
| Chimtom Nwaosu | University College London Hospitals NHS Foundation Trust, London |
| Chris Valerio | North Middlesex University Hospital NHS Trust, London |
| Christine Hosein | University College London Hospitals NHS Foundation Trust, London |
| Chuen Khaw | Lungs for Living Research Centre, UCL Respiratory, University College London, London |
| Claire Levermore | University College London Hospitals NHS Foundation Trust, London |
| Columbus Ife | University College London Hospitals NHS Foundation Trust, London |
| Daryl O. Cheng | Satsuma Lab, UCL Hawkes Institute |
| Derya Ovayolu | University College London Hospitals NHS Foundation Trust, London |
| Dominique Arancon | University College London Hospitals NHS Foundation Trust, London |
| Eleanor Hellier | University College London Hospitals NHS Foundation Trust, London |
| Elena Stefan | University College London Hospitals NHS Foundation Trust, London |
| Elodie Murali | University College London Hospitals NHS Foundation Trust, London |
| Esther Arthur-Darkwa | CRUK & UCL Cancer Trials Centre, University College London, London |
| Ethaar El-Emir | University College London Hospitals NHS Foundation Trust, London |
| Fabia Ruggiero | University College London Hospitals NHS Foundation Trust, London |
| Fahiza Chowdhury | University College London Hospitals NHS Foundation Trust, London |
| Fahmida Hoque | University College London Hospitals NHS Foundation Trust, London |
| Fanta Bojang | University College London Hospitals NHS Foundation Trust, London |
| Farhida Begum | University College London Hospitals NHS Foundation Trust, London |
| Geoff Bellingan | University College London Hospitals NHS Foundation Trust, London |
| Georgia Bullock | University College London Hospitals NHS Foundation Trust, London |
| Graham Robinson | University College London Hospitals NHS Foundation Trust, London |
| Gulistan Keskinbicak | University College London Hospitals NHS Foundation Trust, London |
| Gulten Geneci | University College London Hospitals NHS Foundation Trust, London |
| Harriet Sorrell | University College London Hospitals NHS Foundation Trust, London |
| Hasti Robbie | University College London Hospitals NHS Foundation Trust, London |
| Helen Hall | Lungs for Living Research Centre, UCL Respiratory, University College London, London |
| Helen Kiconco | University College London Hospitals NHS Foundation Trust, London |
| Helen McGregor | Satsuma Lab, UCL Hawkes Institute |
| Helen Victoria Bowyer | University College London Hospitals NHS Foundation Trust, London |
| Henna Makwana | University College London Hospitals NHS Foundation Trust, London |
| Hina Pervez | University College London Hospitals NHS Foundation Trust, London |
| Houda Benmebarek | University College London Hospitals NHS Foundation Trust, London |
| James Rusius | Noclor Research Support, London |
| Jane Rowlands | University College London Hospitals NHS Foundation Trust, London |
| Janine Zylstra | University College London Hospitals NHS Foundation Trust, London |
| Jeannie Eng | University College London Hospitals NHS Foundation Trust, London |
| Jennifer L Dickson | Lungs for Living Research Centre, UCL Respiratory, University College London, London |
| Jhanara Begum | University College London Hospitals NHS Foundation Trust, London |
| John McCabe | Lungs for Living Research Centre, UCL Respiratory, University College London, London |
| Jon Teague | CRUK & UCL Cancer Trials Centre, University College London, London |
| Joseph Jacob | Satsuma Lab, Centre for Medical Image Computing (CMIC), London |
| Judy Airebamen | University College London Hospitals NHS Foundation Trust, London |
| Julian McKee | University College London Hospitals NHS Foundation Trust, London |
| Karen Parry-Billings | University College London Hospitals NHS Foundation Trust, London |
| Karen Sennett | Islington Clinical Commissioning Group, London |
| Kate Davies | Lungs for Living Research Centre, UCL Respiratory, University College London, London |
| Kate Gowers | Lungs for Living Research Centre, UCL Respiratory, University College London, London |
| Kaylene Phua | University College London Hospitals NHS Foundation Trust, London |
| Kim Sorley | University College London Hospitals NHS Foundation Trust, London |
| Kitty Chan | CRUK & UCL Cancer Trials Centre, University College London, London |
| Kylie Gyertson | University College London Hospitals NHS Foundation Trust, London |
| Laila Ahmed | University College London Hospitals NHS Foundation Trust, London |
| Laura Farrelly | CRUK & UCL Cancer Trials Centre, University College London, London |
| Laura Green | University College London Hospitals NHS Foundation Trust, London |
| Lucas Clarke | University College London Hospitals NHS Foundation Trust, London |
| Lynsey Gallagher | University College London Hospitals NHS Foundation Trust, London |
| Magali Taylor | University College London Hospitals NHS Foundation Trust, London |
| Malavika Suresh | CRUK & UCL Cancer Trials Centre, University College London, London |
| Mamta Ruparel | Lungs for Living Research Centre, UCL Respiratory, University College London, London |
| Marie Calligaris | University College London Hospitals NHS Foundation Trust, London |
| Marilyn Ijeomah-Orji | University College London Hospitals NHS Foundation Trust, London |
| Mark Clark | University College London Hospitals NHS Foundation Trust, London |
| Maureen Browne | University College London Hospitals NHS Foundation Trust, London |
| Mehran Azimbagirad | Satsuma Lab, Centre for Medical Image Computing (CMIC), London |
| Miguel Ferreira | University College London Hospitals NHS Foundation Trust, London |
| Milica Rajkov | University College London Hospitals NHS Foundation Trust, London |
| Moksud Miah | University College London Hospitals NHS Foundation Trust, London |
| Monica L. Mullin | Lungs for Living Research Centre, UCL Respiratory, University College London, London  Department of Respirology, University of Calgary, Calgary |
| Monika Sommerrey | University College London Hospitals NHS Foundation Trust, London |
| Navinah Nundlall | University College London Hospitals NHS Foundation Trust, London |
| Nazim Miah | University College London Hospitals NHS Foundation Trust, London |
| Neal Navani | Lungs for Living Research Centre, UCL Respiratory, University College London, London  University College London Hospitals NHS Foundation Trust, London |
| Neil Songsong | University College London Hospitals NHS Foundation Trust, London |
| Niccolo McConnell | Satsuma Lab, UCL Hawkes Institute |
| Nicholas Beech | University College London Hospitals NHS Foundation Trust, London |
| Nick Screaton | Royal Papworth Hospital NHS Foundation Trust, Cambridge |
| Nikita Sharma | University College London Hospitals NHS Foundation Trust, London |
| Olufunmilola Ajayi | University College London Hospitals NHS Foundation Trust, London |
| Oluwadara Akerewusi | University College London Hospitals NHS Foundation Trust, London |
| Pardeep Vasudev | Satsuma Lab, UCL Hawkes Institute |
| Patricia Castello | University College London Hospitals NHS Foundation Trust, London |
| Paul Hickey | University College London Hospitals NHS Foundation Trust, London |
| Paul Robinson | University College London Hospitals NHS Foundation Trust, London |
| Priyam Verghese | Lungs for Living Research Centre, UCL Respiratory, University College London, London |
| Qin Neville | University College London Hospitals NHS Foundation Trust, London |
| Rabiya Patel | University College London Hospitals NHS Foundation Trust, London |
| Rachael Sarpong | CRUK & UCL Cancer Trials Centre, University College London, London |
| Rajesh Banka | Barking, Havering and Redbridge University Hospitals NHS Trust, Essex |
| Ricardo McEwen | University College London Hospitals NHS Foundation Trust, London |
| Robert Chapman | Satsuma Lab, UCL Hawkes Institute |
| Rosie Dapaah Tweneboah | University College London Hospitals NHS Foundation Trust, London |
| Rowan Faiers | University College London Hospitals NHS Foundation Trust, London |
| Ruth Prendecki | Lungs for Living Research Centre, UCL Respiratory, University College London, London |
| Ryoko Egashira | Satsuma Lab, UCL Hawkes Institute |
| Sahana Uthayakumar | University College London Hospitals NHS Foundation Trust, London |
| Sam Hare | University College London Hospitals NHS Foundation Trust, London |
| Sam M Janes | Lungs for Living Research Centre, UCL Respiratory, University College London, London  University College London Hospitals NHS Foundation Trust, London |
| Samanjit Hare | Royal Free London NHS Foundation Trust, London |
| Samantha L Quaife | Centre for Cancer Screening, Prevention, Detection and Early Diagnosis, Wolfson Institute of Population Health, Barts and The London School of Medicine and Dentistry, Queen Mary University of London, London |
| Sara Lock | Whittington Health NHS Trust, London |
| Sebri Hasaballa | University College London Hospitals NHS Foundation Trust, London |
| Shahab Aslani | Satsuma Lab, UCL Hawkes Institute |
| Shaira Hassan | University College London Hospitals NHS Foundation Trust, London |
| Shanshan Wang | Satsuma Lab, UCL Hawkes Institute |
| Sheetal Karavadra | University College London Hospitals NHS Foundation Trust, London |
| Shiyu Wang | Satsuma Lab, UCL Hawkes Institute |
| Shrinkhala Dawadi | CRUK & UCL Cancer Trials Centre, University College London, London |
| Shummi Begum | University College London Hospitals NHS Foundation Trust, London |
| Simranjit Mehta | University College London Hospitals NHS Foundation Trust, London |
| Sofia Nnorom | University College London Hospitals NHS Foundation Trust, London |
| Sophie Tisi | Lungs for Living Research Centre, UCL Respiratory, University College London, London |
| Stephen Ellis | University College London Hospitals NHS Foundation Trust, London |
| Stuart Marnham | University College London Hospitals NHS Foundation Trust, London |
| Sumiya Mahey | University College London Hospitals NHS Foundation Trust, London |
| Suraiya Sharmin | University College London Hospitals NHS Foundation Trust, London |
| Syeda Anam Hussain | University College London Hospitals NHS Foundation Trust, London |
| Sylvia Patricia Enes | University College London Hospitals NHS Foundation Trust, London |
| Tania Anastasiadis | Tower Hamlets Clinical Commissioning Group, London |
| Tanita Limani | University College London Hospitals NHS Foundation Trust, London |
| Tanya Patrick | Lungs for Living Research Centre, UCL Respiratory, University College London, London |
| Terry O'Shaughnessy | Barts Health NHS Trust, London |
| Thea Buchan | University College London Hospitals NHS Foundation Trust, London |
| Theepan Visakan | University College London Hospitals NHS Foundation Trust, London |
| Thomas Callender | Lungs for Living Research Centre, UCL Respiratory, University College London, London |
| Tracy Odigie | University College London Hospitals NHS Foundation Trust, London |
| Trisha Enes | University College London Hospitals NHS Foundation Trust, London |
| Tunku Aziz | Barts Health NHS Trust, London |
| Ugo Ekeowa | The Princess Alexandra Hospital NHS Trust, Essex |
| Urja Patel | University College London Hospitals NHS Foundation Trust, London |
| Vicky Bowyer | University College London Hospitals NHS Foundation Trust, London |
| William M Ricketts | Barts Health NHS Trust, London |
| Ya-Fei Li | University College London Hospitals NHS Foundation Trust, London |
| Yamada Daisuke | Satsuma Lab, UCL Hawkes Institute |
| Yee Chin Lee | University College London Hospitals NHS Foundation Trust, London |
| Zaheer Mangera | North Middlesex University Hospital NHS Trust, London |
| Zahra Awan | University College London Hospitals NHS Foundation Trust, London |
| Zahra Hanif | University College London Hospitals NHS Foundation Trust, London |
| Zelena Aziz | University College London Hospitals NHS Foundation Trust, London |
